# Catastrophic venous and arterial thrombosis in a young female with cervical cancer

**DOI:** 10.1002/jha2.973

**Published:** 2024-07-23

**Authors:** Jordan Burgess, Fraser Hendry, Catherine Bagot, Brian Doherty

**Affiliations:** ^1^ Department of Haematology Beatson West of Scotland Cancer Centre Glasgow UK; ^2^ Department of Radiology Glasgow Royal Infirmary Glasgow UK; ^3^ Department of Haematology Glasgow Royal Infirmary Glasgow UK

1

A 31‐year‐old woman presented with progressive left arm, and neck swelling 2 weeks after a blood transfusion via a cannula in her left antecubital fossa, for severe menorrhagia. Imaging (Figure 1) demonstrated extensive deep vein thrombosis of the left arm extending to the skull base (top left image), extensive bilateral pulmonary emboli, and prominent, subcentimeter para‐aortic and bilateral pelvic lymph nodes. The D‐dimer level was significantly elevated at 46,212 ng/mL (0‒230). She was immediately started on apixaban.

**FIGURE 1:**
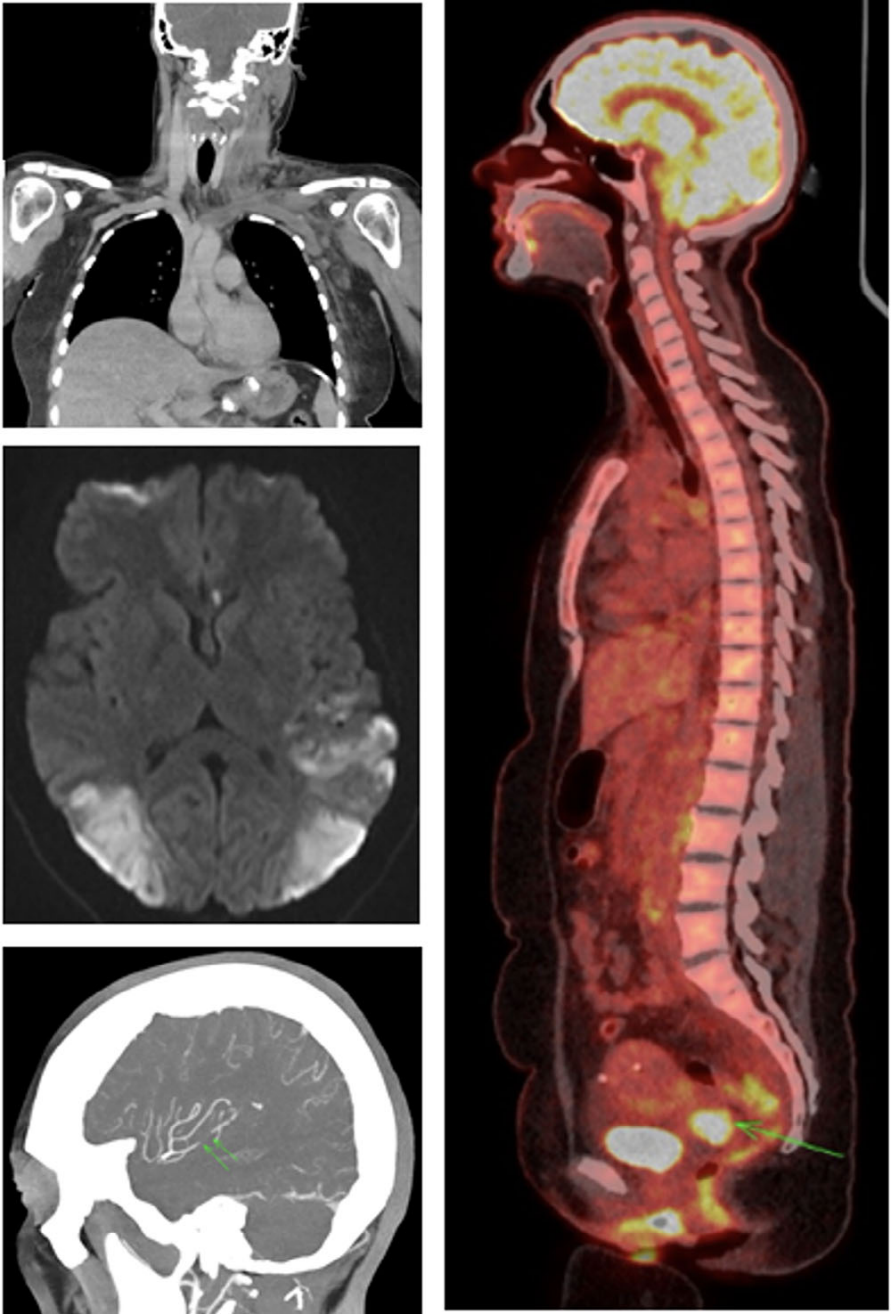
Extensive deep vein thrombosis of the left arm extending to the skull base (top left image). Short segment occlusion of the left middle cerebral artery (bottom left image). Bilateral parieto‐occipital infarction (middle left image). Positron emission tomography (PET) uptake in the cervix (right‐sided image).

Two weeks later, she presented with progressive headache and visual loss and was diagnosed with a left sigmoid sinus thrombus, a short segment occlusion of the left middle cerebral artery (bottom left image) and bilateral parieto‐occipital infarction (middle left image). The strokes manifested as cortical blindness and aphasia. There were no concerns regarding the patient's compliance with apixaban; an anti‐Xa apixaban level confirmed that she had taken a recent dose. The patient was switched to twice daily enoxaparin, aiming for a peak anti‐Xa level of 1.0‒1.2 U/mL. Aspirin 75 mg daily was also initiated.

She was urgently investigated for possible causes of this severe prothrombotic state, including catastrophic anti‐phospholipid syndrome, thrombotic thrombocytopenic purpura, myeloproliferative neoplasms, paroxysmal nocturnal hemoglobinuria, and auto‐immune heparin‐induced thrombocytopenia, all of which were negative. A further total body computed tomography demonstrated no change in the lymph node features but revealed new splenic and renal infarcts. On transthoracic echocardiogram, a thrombus was visible on both the tricuspid and mitral valves. In the absence of an identifiable cause, positron emission tomography was performed, demonstrating uptake in the cervix (right‐sided image), para‐aortic lymph nodes and peritoneal deposits. A cervical biopsy confirmed a diagnosis of metastatic cervical adenocarcinoma that was positive for high‐risk human papillomavirus (HPV)45. Interestingly, cervical screening was HPV negative 20 months prior to this presentation. The patient unfortunately died shortly after commencing palliative chemotherapy.

Cancer is a hypercoagulable state associated with a sevenfold increase in venous thromboembolism; however, the association with arterial thromboembolism is less well‐established [1]. Mucin‐producing adenocarcinomas are one of the most common tumours associated with venous thromboembolism (VTE) [2] since mucin directly stimulates platelet activation [3]. Patients with cervical cancer have a higher cumulative risk of VTE as compared to the general population [4]. The incidence of thromboembolism has been demonstrated to be highest during chemotherapy [5].

## AUTHOR CONTRIBUTIONS

Jordan Burgess wrote the paper. Fraser Hendry supplied images and performed interpretation of radiological findings. Catherine Bagot helped in writing the paper and was consultant in charge of patient. Brian Doherty helped in writing the paper.

## CONFLICT OF INTEREST STATEMENT

The authors declare they have no conflicts of interest regarding the publication of this manuscript.

## FUNDING INFORMATION

The authors received no specific funding for this work.

## ETHICS STATEMENT

The authors have confirmed that an ethical approval statement is not needed for this submission.

## PATIENT CONSENT STATEMENT

The authors have obtained a patient consent statement for this submission.

## PERMISSION TO PRODUCE MATERIAL FROM OTHER SOURCES

N/A.

## CLINICAL TRIAL REGISTRATION

The authors have confirmed that clinical trial registration is not needed for this submission.

## Data Availability

The data that support the findings of this study are available upon request from the corresponding author. The data are not publicly available due to privacy or ethical restrictions.
