# Correction to “Catastrophic Venous and Arterial Thrombosis in a Young Female With Cervical Cancer”

**DOI:** 10.1002/jha2.70149

**Published:** 2025-09-24

J. Burgess, F. Hendry, C. Bagot, and B. Doherty, “Catastrophic Venous and Arterial Thrombosis in a Young Female With Cervical Cancer,” *eJHaem* 5, no. 4 (2024): 879–880, https://doi.org/10.1002/jha2.973.

Email address for Jordan Burgess has been removed from original article due to phishing activity.

We apologise for this error.

